# 1911. A Pilot Study to Evaluate the Effectiveness of Nasal and Oral Povidone Iodine in Reducing the Burden of Severe Acute Respiratory Syndrome 2 RNA in Patients with COVID-19

**DOI:** 10.1093/ofid/ofac492.1538

**Published:** 2022-12-15

**Authors:** Sarah Redmond, Lucas D Jones, Alexandria Nguyen, Hussein Ghaddara, Jennifer Cadnum, Curtis Donskey

**Affiliations:** Case Western Reserve Medical School, Cleveland, Ohio; Case Western Reserve University School of Medicine, Cleveland Heights, Ohio; Northeast Ohio VA Healthcare System, Cleveland, Ohio; Northeast Ohio VA Healthcare System, Cleveland, Ohio; Northeast Ohio VA Medical Center, Cleveland, Ohio; Cleveland VA Hospital, Cleveland, Ohio

## Abstract

**Background:**

Nasal and oral application of topical antiseptics such as povidone iodine could potentially reduce the risk for transmission of severe acute respiratory syndrome coronavirus 2 (SARS-CoV-2). However, limited information is available on the efficacy of such agents in reducing the burden of SARS-CoV-2.

**Methods:**

We conducted a pilot non-blinded, randomized trial to compare the effectiveness of 3 doses of povidone iodine (each dose with 10% intranasal and 1% gargle) administered every 8 hours versus the control with phosphate-buffered saline in reducing the burden of SARS-CoV-2 RNA in the nares and oropharynx of patients with COVID-19. Swabs were used to collect anterior nares and oropharynx samples before the first and second doses and 8 hours after the final dose (24 hours after the initial dose). Real-time polymerase-chain reaction (RT-PCR) was used to assess the burden of viral RNA. Analysis of variance was used to compare cycle threshold values for povidone iodine versus control patients. Subjects were surveyed about adverse reactions to treatment.

**Results:**

As shown in the figure, SARS-CoV-2 cycle thresholds were similar in the povidone iodine (N=10 subjects) and control (N=8 subjects) groups prior to treatment. After initiation of treatment, there was no significant difference in cycle thresholds for the povidone iodine versus control subjects (P >0.05). No adverse effects of treatment were reported.

Effect of intranasal and oral application of povidone iodine versus phosphate-buffered saline on nasal and oropharyngeal SARS-CoV-2 RNA. Error bars show standard error.

**Figure:**
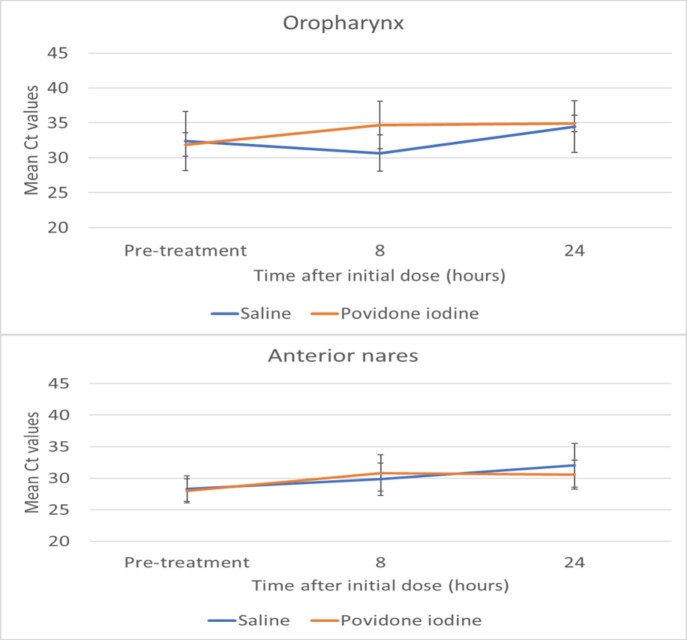

**Conclusion:**

Our findings suggest that that nasal and oral application of povidone iodine have limited effectiveness in reducing the burden of SARS-CoV-2. Future studies are needed to assess for effectiveness of more frequent dosing intervals and to determine if povidone iodine reduces recovery of viable virus by culture.

**Disclosures:**

**All Authors**: No reported disclosures.

